# Quantitative autism symptom patterns recapitulate differential mechanisms of genetic transmission in single and multiple incidence families

**DOI:** 10.1186/s13229-015-0050-z

**Published:** 2015-10-27

**Authors:** Thomas W. Frazier, Eric A. Youngstrom, Antonio Y. Hardan, Stelios Georgiades, John N. Constantino, Charis Eng

**Affiliations:** Center for Autism (CRS10), Pediatric Institute, Cleveland Clinic, 2801 Martin Luther King Jr. Drive, Cleveland, OH 44104 USA; Department of Psychology, University of North Carolina at Chapel Hill, 235 E. Cameron Avenue, Davie Hall CB #3270, Chapel Hill, NC 27599-3520 USA; Department of Psychiatry and Behavioral Science, Stanford University, 401 Quarry Road, Stanford, CA 94305-5717 USA; Department of Psychiatry and Behavioural Neurosciences, Offord Centre for Child Studies, McMaster University, 555 Sanatorium Rd, Hamilton, ON L9C 2B1 Canada; Department of Psychiatry, Washington University of St. Louis, 660 S. Euclid Avenue, St. Louis, MO 63110 USA; Genomic Medicine Institute, Taussig Cancer Institute, and Stanley Shalom Zielony Institute of Nursing Excellence, Cleveland Clinic, 9500 Euclid Avenue, Cleveland, OH 44195 USA

**Keywords:** Autism spectrum disorder, Multiple incidence families, Genetic epidemiology, Autism symptoms, DSM-5

## Abstract

**Background:**

Previous studies have demonstrated aggregation of autistic traits in undiagnosed family members of children with autism spectrum disorder (ASD), which has significant implications for ASD risk in their offspring. This study capitalizes upon a large, quantitatively characterized clinical-epidemiologic family sample to establish the extent to which family transmission pattern and sex modulate ASD trait aggregation.

**Methods:**

Data were analyzed from 5515 siblings (2657 non-ASD and 2858 ASD) included in the Interactive Autism Network. Autism symptom levels were measured using the Social Responsiveness Scale (SRS) and by computing Diagnostic and Statistical Manual of Mental Disorders—Fifth Edition (DSM-5) symptom scores based on items from the SRS and Social Communication Questionnaire. Generalized estimating equation models evaluated the influence of family incidence types (single versus multiple incidence families; male-only ASD-affected families versus families with female ASD-affected children), diagnostic group (non-ASD children with and without a history of language delay with autistic speech and ASD-affected children), and sibling sex on ASD symptom levels.

**Results:**

Non-ASD children manifested elevated ASD symptom burden when they were members of multiple incidence families—this effect was accentuated for male children in female ASD-containing families—or when they had a history of language delay with autistic qualities of speech. In this sample, ASD-affected children from multiple incidence families had lower symptom levels than their counterparts in single incidence families. Recurrence risk for ASD was higher for children from female ASD-containing families than for children from male-only families.

**Conclusions:**

Sex and patterns of family transmission modulate the risk of autism symptom burden in undiagnosed siblings of ASD-affected children. Identification of these symptoms/traits and their molecular genetic causes may have significant implications for genetic counseling and for understanding inherited liabilities that confer risk for ASD in successive generations. Autism symptom elevations were more dramatic in non-ASD children from multiple incidence families and those with a history of language delay and autistic qualities of speech, identifying sub-groups at substantially greater transmission risk. Higher symptom burden and greater recurrence in children from female ASD-containing families indicate that familial aggregation patterns are further qualified by sex-specific thresholds, supportive of the notion that females require a higher burden of deleterious liability to cross into categorical ASD diagnosis.

**Electronic supplementary material:**

The online version of this article (doi:10.1186/s13229-015-0050-z) contains supplementary material, which is available to authorized users.

## Background

Autism spectrum disorder (ASD) is a phenotypically and etiologically heterogeneous neurodevelopmental disorder [1] with common core features of social communication/interaction impairment and the presence of restricted/repetitive behaviors [2]. Behavioral genetic studies have identified a strong genetic component [3, 4], derived from a wide range of genomic mechanisms [5], including possible gene-environment interactions. Family-based studies of autism symptoms have found evidence of inter-generational transmission via familial aggregation of elevated scores. At least a subset of unaffected family members from multiple incidence families, most often siblings and fathers [6], have greater social cognitive [7, 8] and autism trait burden [9–11], and parents with subtle elevations of autism traits are at greater risk of having an ASD-affected child [12].

Recent genetic studies support phenotypic findings, identifying greater additive genetic burden in multiple incidence families [13]. However, the exact pattern of elevated symptom burden in non-ASD children from single and multiple incidence families remains uncertain. This leads to several important questions. Do elevated symptom levels in non-ASD children from multiple incidence families vary across different autism symptom domains, as would be predicted from studies implying different etiological influences on social and repetitive behavior symptoms [14, 15]? Or is a general predisposition observed toward social deficits and restricted/repetitive behavior [3]? Do ASD-affected children from multiple incidence families also show a more severe symptom burden relative to their counterparts from single incidence families? Answers to these questions will provide important information about autism transmission risk and may provide clues to differences in the mixtures of etiologic mechanisms between these incidence patterns. Furthermore, unaffected siblings who have a history of language delay with autistic qualities of speech (HLDAS) have greater autism symptom burden [9]. Examining HLDAS in both single and multiple incidence families is important for determining whether increased etiologic burden in unaffected siblings extends beyond multiple incidence families to a subset of single incidence families and whether HLDAS further amplifies risk.

Family-based studies can also be helpful for understanding how sex of the affected child influences autism symptom levels. The best known risk factor for autism is being male [16], and increasingly disparate sex ratios are observed at the higher functioning end of the autism spectrum [17–19]. An early study focusing on categorical ASD diagnoses in families did not suggest increased genetic loading or sex-specific thresholds in families containing ASD-affected females (female ASD-containing families) [20]. However, more recently, a study of male-only and female ASD-containing families found evidence of higher thresholds for restricted/repetitive behavior in females [21]. Additionally, using the Autism Genetic Resource Exchange cohort, Werling and Geschwind identified higher recurrence rates in siblings of female probands [22]. Studies of copy number variation have further supported the idea of sex-specific thresholds, identifying a higher mutational burden in ASD-affected females [23] and differential genetic and hormonal factors that may contribute to sex-specific thresholds [24]. No previous studies have investigated whether increased phenotypic burden in female ASD-containing families extends to both non-ASD and ASD-affected children, if the pattern differs across single and multiple incidence families or if autism symptom measurements yield distinct patterns across specific Diagnostic and Statistical Manual of Mental Disorders—Fifth Edition (DSM-5) symptom criteria.

Using the large Interactive Autism Network (IAN) database, the present study investigated these three major factors (single vs. multiple incidence families, HLDAS in unaffected children, and male-only vs. female ASD-containing families) to provide a more detailed picture of genetic burden and transmission risk. Analyses first focused on autism symptom levels in non-ASD and ASD-affected children from single and multiple incidence families. We anticipated that ASD-affected children from multiple incidence families would have lower symptom levels than their counterparts from single incidence families, opposite the pattern of non-ASD children [9], implying a unique etiologic mixture rather than greater overall symptom and genetic burden. Next, we examined whether a history of language delay with autistic qualities of speech in non-ASD siblings resulted in increased symptom burden. We expected that non-ASD children with HLDAS would show higher symptom levels than non-ASD children without HLDAS and that HLDAS would add to the enrichment expected for multiple incidence families. Finally, we evaluated whether the sex of the ASD-affected child (male-only versus female ASD-containing families) further modified autism symptom levels. Non-ASD children from female ASD-containing families were predicted to show higher symptom levels than their counterparts from male-only families and recurrence risk was expected to be higher for next-born siblings in female ASD-containing families.

## Methods

### Participants

Data were obtained from the IAN (http://www.ianproject.org), an Internet-based registry for families with one or more ASD-affected children (Data Export ID IAN_DATA_2013-01-28). Families were eligible for enrollment in IAN if the parent or legal guardian who provided information was English speaking, the family lived in the USA, and their child was diagnosed with ASD by a professional. To be included in the present study, caregivers must have reported data for at least one ASD-affected child and at least one additional child. To evaluate symptom levels across child and family characteristics, a subset of the IAN registry was used with complete data on both the Social Communication Questionnaire (SCQ) and the Social Responsiveness Scale (SRS). Previous analyses have shown that this subset is highly similar to the larger IAN registry with a few exceptions: the SRS/SCQ subset is older due to SRS administration beginning at age 4, has fewer non-verbal individuals likely as a result of several SRS items tapping the use of language, and has a greater proportion of white/non-Hispanic youth [25]. Each of these differences was found to be very small in magnitude (*r* < .10).

### Procedures

Informed consent was obtained from parents/guardians for all participants prior to entry into the IAN data collection. The procedures of IAN and the present study were reviewed and approved by the institutional review boards of Kennedy Krieger Institute and Cleveland Clinic, respectively.

Demographics, clinical diagnoses, and autism symptoms were provided by caregivers as part of participation in IAN. Demographics included: age, sex, and race/ethnicity (coded as white non-Hispanic and other race/ethnicity). Each family was coded for two characteristics: (1) family incidence type and (2) family sex type. To code families, an index child was first identified by locating the oldest ASD-affected child associated with each unique IAN family identifier. Single incidence families were coded if at least one non-ASD (unaffected) child and no other ASD-affected children beyond the index case were associated with the same IAN family identifier. Multiple incidence families were coded if at least one additional ASD-affected child beyond the index case was associated with the same IAN family identifier. Female ASD-containing families were coded if at least one female ASD-affected child and possibly other male ASD-affected children were present. Male-only families were coded if all ASD-affected children in the family were males. Parent and other relative diagnoses were not consistently available in IAN and were not considered in family sex-type coding. Family sex-type definitions followed previous conventions [21, 22] and, in both types of families, non-ASD female and male children are possible. Recurrence risk was calculated separately for next-born male and female siblings and for female ASD-containing and male-only families. These calculations used the diagnostic classification (ASD vs. non-ASD) for the oldest available sibling after the index case in all families [22].

#### ASD diagnosis

Caregivers reported whether each registered child was affected by ASD and, if affected, the specific DSM-IV-TR diagnosis obtained from a previous clinical evaluation. In the SRS/SCQ subset of IAN, the vast majority of clinical ASD diagnoses (93 %) were provided by a doctoral level professional or team. A substantial proportion of youth (74.9 %) was diagnosed using the Autism Diagnostic Interview—Revised, the Autism Diagnostic Observation Schedule, or both. Of these, 98.7 % scored in the ASD-affected range for one or both instruments. Recent study of youth from the IAN registry provided additional support for the validity of clinical ASD diagnoses [26, 27]. Importantly, randomly selected verbal youth with a score ≥12 on the SCQ were evaluated using the Autism Diagnostic Interview—Revised and by expert clinician observation. All but a handful of youth (98 %) were confirmed to have a DSM-IV-TR clinical diagnosis of ASD. ASD was coded by collapsing all DSM-IV-TR autism spectrum diagnoses into a single category consistent with the Center for Disease Control epidemiological catchment protocols. A substantial minority of the siblings registered as not having an ASD diagnosis have caregiver-reported psychiatric or developmental diagnoses (ADHD 12 %, a history of motor delay 7 %, an anxiety disorder 6 %, mood disorder 4 %, and intellectual disability 0.5 %). For this reason and to be consistent with previous publications, we refer to these siblings as non-ASD children [25]. Previous data have suggested that a subset of unaffected children from ASD-affected families carry a specific diathesis toward elevated autism traits [9]. On this basis, non-ASD children were further divided into those with a history of language delay and autistic qualities of speech (non-ASD with HLDAS) and those without (non-ASD) to investigate whether this distinction identifies children at high risk of future genetic transmission. HLDAS was coded as positive if any of SCQ items 3–7 were endorsed consistent with a previous study [9].

#### Autism symptoms

Autism symptom constructs were obtained in two ways. The first used the total and sub-scales of the SRS [28]. The SRS is 65-item, ordinally scaled (1 = “not true” to 4 = “almost always true”) quantitative assessment of the severity of autism symptoms. The SRS has been extensively validated and distinguishes youth with autism from other psychiatric conditions [11, 28]. The present study used SRS total raw scores to examine family incidence type and family sex type. In addition to the total score, SRS raw sub-scale scores were computed as item averages for the five factor analytically derived scales: emotion recognition, social avoidance, relationships, repetitive sensory motor, and insistence on sameness [29].

#### DSM-5 symptoms

DSM-5 symptom scores were computed using items from the SCQ and SRS that were mapped to these criteria [25, 30] (Additional file 1). For each DSM-5 symptom, counts were obtained by summing SCQ and SRS items mapped to that symptom and then dividing by the total possible score. This process yielded a single, comparable metric for each of the seven DSM-5 symptoms, ranging from 0 (no endorsement) to 1 (all items endorsed). Internal consistency reliability was very good for brief scales (*α* = .73–.91), indicating that any differences in findings across DSM-5 symptom scales are not due to differential reliability.

### Analytic plan

Independent samples *t* tests and Pearson’s chi-squared test were used to compare sample characteristics across single and multiple incidence families. When non-normal distributions were observed, non-parametric equivalent statistics were performed and produced the same pattern of results. To facilitate effect size comparison, parametric statistics were reported.

To estimate symptom level differences between single and multiple incidence families, generalized estimating equation (GEE) models included individual- and family-level fixed effects factors: family incidence type (single versus multiple incidence), diagnostic group (ASD versus non-ASD with and without HLDAS), sex of the child, and their interactions. Age was also included as a fixed effects covariate. SRS total raw scores, factor analytically derived SRS sub-scale scores, and DSM-5 mapped symptom scores were dependent variables in separate analyses. In each model, children were clustered within families. The two-way family incidence type by diagnostic group interaction tested whether autism symptom levels differed across single and multiple incidence families. The three-way interaction adding sex of the child evaluated whether this effect differed in male and female children. To explore whether autism symptom levels were further modified by male-only families vs. female ASD-containing families, a similar model was computed to those above except that family sex type was included as a family-level fixed effects factor, and models were conducted only for non-ASD siblings. In ASD-affected children, a simpler model was computed with the main effects for family incidence type, family sex type, and sex of the child due to the fact that affected females were not present in male-only families. Independent sample *t* tests, means, and confidence intervals were used to describe the pattern of results for family sex type in ASD-affected children. SRS total raw score was the dependent variable in each analysis.

GEE models were estimated using maximum likelihood, and fit was considered by iteratively examining alternative covariance structures and link functions [31]. Final models were presented based on unstructured covariance structure and a linear link function, which fit comparably and yielded a highly similar pattern of results to a Poisson log-linear link function and other covariance structures (e.g., independent, autoregressive, and exchangeable). Age and sex of the child were included in all analyses of SRS raw scores and DSM-5 symptom scores to ensure these factors did not confound interpretation of the effect of family characteristics on autism symptom levels.

To further characterize the meaning of elevated symptom levels in non-ASD children, follow-up analyses computed Pearson’s correlations estimating bivariate relationships between autism symptoms and developmental milestones: age of independent walking, age of first meaningful phrase speech, and presence of motor delay. Additionally, differences in developmental milestones between non-ASD siblings with and without HLDAS were computed using analysis of variance for age of walking and age of first use of meaningful phrase speech and chi-squared analysis for motor delay.

All analyses were computed in IBM SPSS version 23. Statistical significance was determined using *p* < .05. False discovery rate corrections were applied when multiple autism symptom measures were examined to control for type 1 error inflation due to multiple testing [32]. Given the large sample size and strong power to detect small covariate effects (power > .80 for partial *r*_ab.c_ = .04 and larger), the pattern and magnitude of effects across autism symptom measures were also considered in interpretation. Single degree of freedom *F* and *X*^2^ statistics were converted to Cohen’s *d* [33]. Cohen’s *d* values ≤.20 and .20–.50 were considered small and small-to-medium effects, respectively [34].

## Results

### Sample description

Children from single and multiple incidence families did not significantly differ on age, race/ethnicity, or the presence of DSM-IV-TR diagnoses of autistic disorder or pervasive developmental disorder not otherwise specified (Table 1). Interestingly, multiple incidence families had a higher proportion of ASD-affected children who received a diagnosis of Asperger’s disorder (21.0 %) relative to single incidence families (15.4 %).

**Table 1:** Sample characteristics of children with autism symptom questionnaire data from single and multiple incidence families in IAN

	Single incidence families	Multiple incidence families	*Χ* ^2^, *t*(*p*)
Number of families	2262	315	
Female ASD-containing (*N*, %)	334 (14.8 %)a	125 (39.7 %)b	*Χ* ^2^(1) = 117.3 (*p* < .001)
Male-only (*N*, %)	1928 (85.2 %)a	190 (60.3 %)b	
Number of children	4764	751	
ASD (*N*, %)	2262 (48.8 %)a	596 (80.6 %)b	*Χ* ^2^(2) = 261.1 (*p* < .001)
Non-ASD (*N*, %)	2097 (45.2 %)a	121 (16.4 %)b	
Non-ASD with HLDAS	280 (6.0 %)a	22 (3.0 %)b	
Age (*M*, SD, range)	8.7 (3.8, 4–18)	8.8 (3.6, 4–18)	*t*(5513) = −0.6 (*p* = .522)
Female siblings (*N*, %)	1602	221	
ASD (*N*, %)	333 (20.8 %)a	141 (63.8 %)b	*Χ* ^2^(2) = 187.7 (*p* < .001)
Non-ASD with HLDAS (*N*, %)	118 (7.4 %)	11 (5.0 %)	
Non-ASD (*N*, %)	1151 (71.8 %)a	69 (31.2 %)b	
Race/ethnicity			
White non-Hispanic	4248 (89.2 %)	684 (91.1 %)	*Χ* ^2^(1) = 2.5 (*p* = .114)
Other or unknown	516 (10.8 %)	67 (8.9 %)	
SCQ total raw (*M*, SD)	12.4 (11.4)	17.5 (10.3)	*t*(5513) = −11.54 (*p* < .001)
SRS total *T* score (*M*, SD)	64.7 (23.8)	77.1 (21.2)	*t*(5513) = −13.47 (*p* < .001)
DSM-IV-TR (*N*, %)			
Autistic disorder	950 (42.0 %)	239 (40.1 %)	*Χ* ^2^(2) = 10.7 (*p* = .005)
PDD NOS	963 (42.6 %)	232 (38.9 %)	
Asperger’s disorder	349 (15.4 %)a	125 (21.0 %)b	

*N* = 137 non-ASD siblings could not be coded into HLDAS due to the missing data regarding the presence of language delay. The numbers of children in each category represent the number of children with complete symptom data and not the total number of children in the family. The numbers for DSM-IV-TR reflect only those with a specific DSM-IV-TR reported diagnosis. A small proportion of ASD-affected individuals did not have a specific DSM-IV-TR diagnosis reported (ASD or PDD was listed instead of a specific diagnosis). Lowercase letters a and b represent column proportions that are significantly different (Bonferroni adjusted *p* < .05). Age is presented as the child’s age when the SRS was completed

### Single versus multiple incidence families

Reliable differences in autism symptom levels were seen between children from single and multiple incidence families (Table 2; smallest *p* = .009 for DSM-5 B2: resistance to change) with all measures surviving false discovery rate correction. The only exception was DSM-5 A2: non-verbal communication where only a trend was observed (*p* = .051). Additional file 2 presents the full analytic results for SRS total raw scores.

**Table 2:** Autism symptom levels in non-ASD, non-ASD with HLDAS, and ASD-affected children by family incidence type

	Non-ASD siblings		Non-ASD siblings with HLDAS		ASD siblings		Family incidence type by diagnostic group
	Single incidence families	Multiple incidence families	Single incidence families	Multiple incidence families	Single incidence families	Multiple incidence families	*Χ* ^2^ (*p*)
	*M* (SD)	*M* (SD)	*M* (SD)	*M* (SD)	*M* (SD)	*M* (SD)	
SRS							
Total raw score	19.7 (19.4)	27.9 (27.3)	41.4 (31.4)	45.5 (25.6)	105.6 (29.0)	101.0 (33.1)	17.28 (<.001)
Emotion recognition	.41 (.37)	.56 (.52)	.83 (.56)	.87 (.44)	1.85 (.48)	1.79 (.55)	14.45 (.001)
Social avoidance	.19 (.31)	.27 (.42)	.35 (.45)	.45 (.51)	1.16 (.62)	1.08 (.65)	9.28 (.010)
Interpersonal relationships	.23 (.39)	.38 (.55)	.57 (.67)	.72 (.71)	1.69 (.66)	1.59 (.71)	11.26 (.004)
Repetitive sensory motor	.19 (.26)	.25 (.31)	.45 (.45)	.48 (.36)	1.35 (.60)	1.26 (.65)	18.11 (<.001)
Insistence on sameness	.29 (.33)	.42 (.46)	.60 (.52)	.66 (.45)	1.63 (.51)	1.56 (.55)	16.75 (<.001)
DSM-5 SCI							
A1: socio-emotional reciprocity	.08 (.12)	.12 (.14)	.19 (.20)	.19 (.16)	.61 (.21)	.59 (.24)	14.20 (.001)
A2: non-verbal communication	.15 (.14)	.16 (.15)	.27 (.19)	.29 (.16)	.64 (.19)	.61 (.21)	5.97 (.051)
A3: relationships	.07 (.14)	.11 (.18)	.20 (.24)	.23 (.23)	.68 (.24)	.63 (.27)	11.80 (.003)
DSM-5 RRB							
B1: repetitive motor	.03 (.10)	.05 (.13)	.09 (.18)	.08 (.18)	.60 (.30)	.53 (.32)	21.74 (<.001)
B2: resistance to change	.13 (.17)	.19 (.24)	.26 (.23)	.30 (.22)	.65 (.24)	.64 (.24)	9.43 (.009)
B3: restricted interests	.06 (.14)	.11 (.16)	.17 (.22)	.17 (.19)	.66 (.25)	.62 (.27)	18.96 (<.001)
B4: abnormal sensory	.05 (.11)	.08 (.13)	.15 (.21)	.12 (.16)	.53 (.25)	.49 (.27)	17.96 (<.001)

Higher raw and *T* scores indicate higher levels of autism traits. Factor analytically derived SRS sub-scales are presented as raw item averages (range 0–3; 0 = not true, 1 = sometimes true, 2 = often true, 3 = almost always true). *T* scores are not currently available for these sub-scales. Each DSM-5 symptom score represents the proportion of endorsed items mapped to that symptom. Scale endpoints represent: 0 = no items endorsed by any individual and 1 = all items endorsed across all individuals

**Fig. 1:**
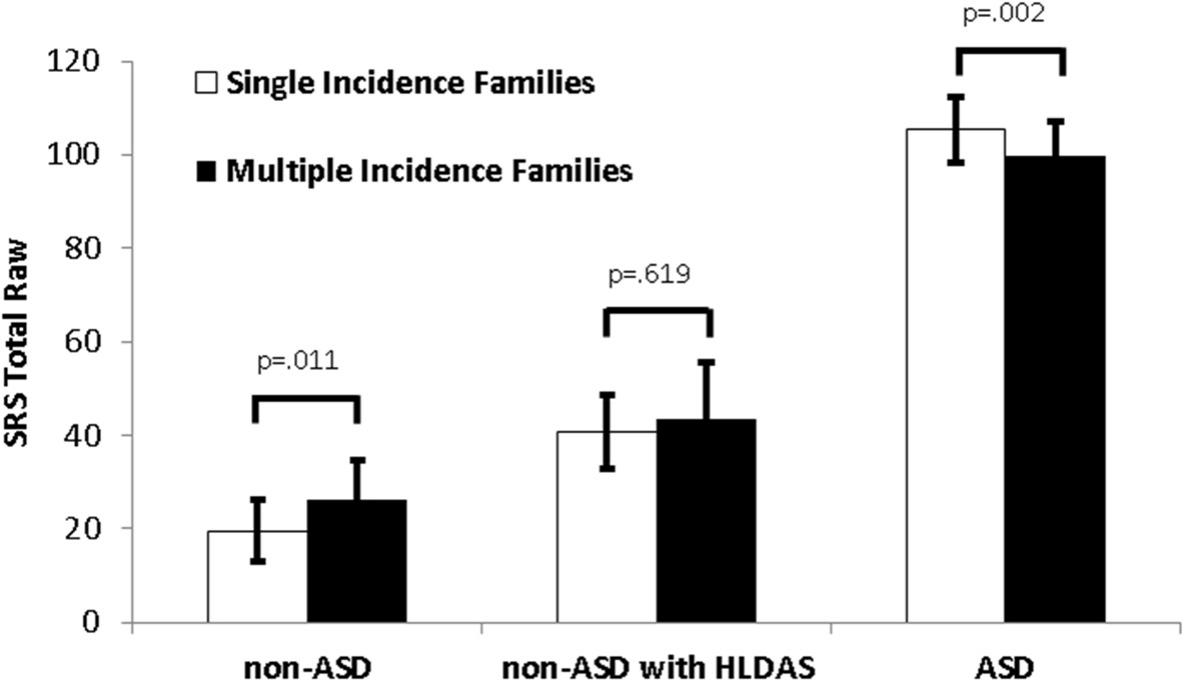
Autism symptoms (*M* +/− 95 % CI) in non-ASD, non-ASD with HLDAS, and ASD-affected children from single and multiple incidence families. *HLDAS* History of language delay with autistic speech

**Fig. 2:**
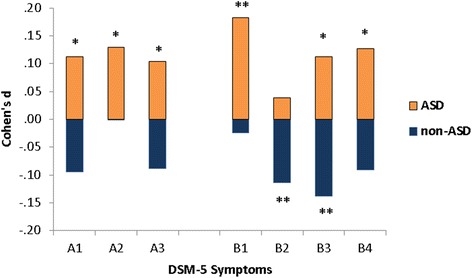
Effect sizes (Cohen’s *d*) representing differences between single and multiple incidence families across DSM-5 symptom domains, separately for ASD and non-ASD children. *p < .05; **p < .01. Positive effect sizes represent higher symptom levels in youth from single incidence families (*orange bars*). Negative effect sizes represent higher symptom levels in youth from multiple incidence families (*light blue bars*)

Non-ASD children without HLDAS from multiple incidence families had higher levels of autism symptoms on the SRS relative to non-ASD children without HLDAS from single incidence families (Fig. 1). This pattern was also generally true for DSM-5 symptoms but the size and significance of effects was variable; significant differences were only observed for DSM-5 B2: resistance to change and B3: restricted interests (Fig. 2). Conversely, ASD-affected children from single incidence families had significantly higher autism symptoms (Fig. 1) than ASD-affected children from multiple incidence families. The effect was largest for B1: repetitive motor (Fig. 2). Inspection of scores indicates that effects were small, but not trivial, with SRS total raw score differences of 4–8 points between children from single and multiple incidence families. As a result, frequency distributions of SRS total raw scores were farther apart in non-ASD and ASD-affected children from single incidence families and closer together in multiple incidence families (Additional file 3 presents the SRS total raw score distributions). Females from multiple incidence families show a distinctly bimodal distribution, replicating previous findings regarding sex-specific aggregation patterns in multiple incidence families [35].

In all non-ASD children, higher autism symptom level was significantly and positively related to age of walking independently, age of first meaningful phrase speech, and the presence of a motor delay (*r* = .06–.21, *p* < .002), supporting the developmental relevance of observed symptom elevations.

### Non-ASD children with HLDAS

Non-ASD children with HLDAS had higher levels of autism symptoms on the SRS (Fig. 1) and higher levels of DSM-5 symptoms (Table 2) relative to non-ASD children without HLDAS (all *p* values <.001). These effects were generally medium to large in magnitude (Cohen’s *d* = .25–.84). Non-ASD children with HLDAS also showed significant delays in developmental milestones relative to non-ASD children without HLDAS, including walking independently (14.4 vs. 12.0 months; *F*(1,2361) = 48.45, *p* < .001), age of first meaningful phrase speech (21.0 vs. 14.5 months; *F*(1,2414) = 85.19, *p* < .001), and the presence of a motor delay (25.8 vs. 3.1 %; *X*^2^(1) = 252.18, *p* < .001). Non-ASD children with HLDAS from multiple incidence families had only slightly higher autism symptom levels relative to non-ASD children with HLDAS from single incidence families, suggesting HLDAS and family type do not result in additive increases in risk of genetic transmission.

### Male-only versus female ASD-containing families

**Fig. 3:**
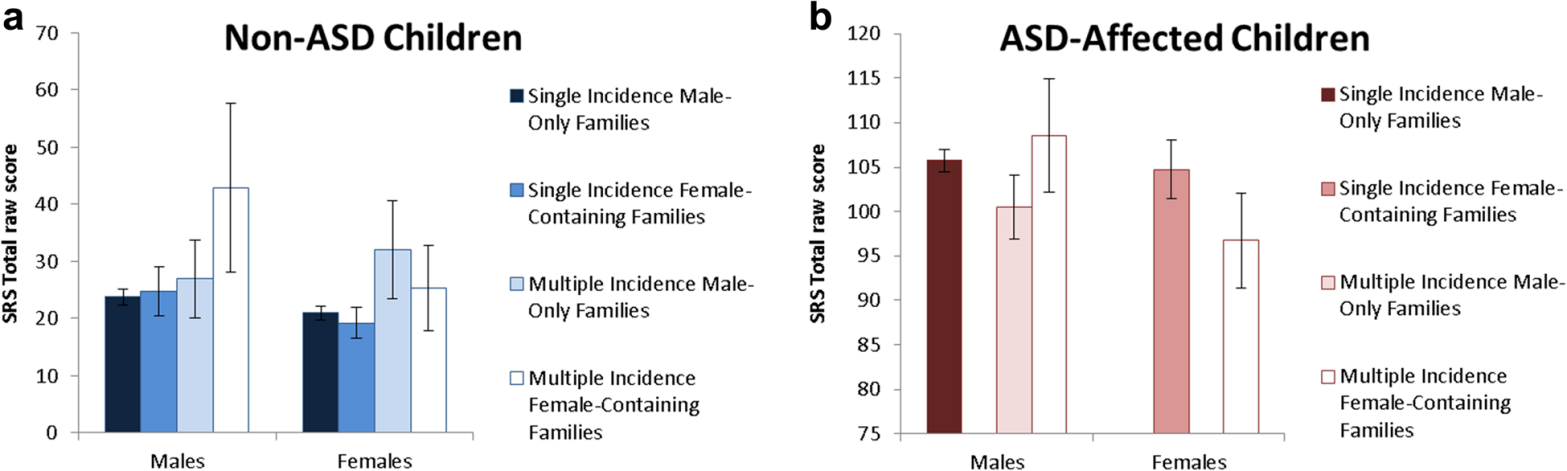
Autism symptom levels (*M* +/− 95 % CI) in female ASD-containing and male-only families, separately for non-ASD (*left panel*) and ASD-affected children (*right panel*)

Lumping non-ASD male and female siblings together, there was no increase in autism symptom burden in female ASD-containing versus male-only families (*X*^2^(1) = 0.60, *p* = .439; Additional file 4). However, non-ASD males from multiple incidence female ASD-containing families had significantly elevated autism symptom levels relative to other non-ASD children (≥11 SRS total raw score points higher) (*X*^2^(1) = 6.74, *p* = .009; Fig. 3a; Additional file 5 presents the full analytic results in non-ASD children). All post hoc comparisons between non-ASD males from multiple incidence female ASD-containing families were significant (*p* ≤ .037), with the exception of only a weak trend for non-ASD female children from multiple incidence male-only families (*p* = .295).

ASD-affected children from female ASD-containing families had significantly higher autism symptom levels than ASD-affected children from male-only families (Additional file 6). Inspection of means and confidence intervals suggests that this effect was driven by higher autism symptom levels in ASD-affected males from multiple incidence female ASD-containing families relative to other ASD-affected children from multiple incidence families (≥8 SRS total raw score points higher) (smallest *t*(453) = 2.16, *p* = .031; Fig. 3b). ASD-affected males and females from single incidence families had comparable symptom levels.

Congruent with well-documented sex rations in ASD, recurrence risk was significantly higher for next-born males (22.0 %) than next-born females (8.6 %) (RR = 2.56, 95 % confidence interval (CI) = 2.15–3.07). More interestingly, recurrence of ASD in female ASD-containing families (31.6 %) was nearly three times higher than in male-only families (11.9 %) (RR = 2.66, 95 % CI = 2.29–3.09) (Additional file 7).

## Discussion

The present study replicated a growing literature showing greater autism symptom burden in non-ASD family members from multiple incidence families [7–9, 12, 36, 37] and generated four unique findings not previously described. First, non-ASD children from multiple incidence families had a *specific pattern* of increased DSM-5 symptom burden. In these children, symptom increases were strongest for behaviors that are prominent in cognitively able individuals with autism (B2: resistance to change, B3: restricted interests). This pattern cannot be explained by differential reliability of scores and instead may indicate that B2 and B3 symptoms are more sensitive to broad ASD-like phenotypic presentations. The pattern may also imply the presence of increased burden of inherited liabilities in multiple incidence families, such as common variation in genes important for the functioning of cortico-striatal-thalamic circuits influencing the manifestation of repetitive/perseverative behavior. In contrast to non-ASD children, ASD-affected children from single incidence families showed increased levels of DSM-5 repetitive motor symptoms associated with cognitively impaired presentations of ASD. This may indicate a disproportionate representation of severely affected individuals in single incidence families, possibly due to strong, de novo genetic variants (large copy number variations (CNVs), multiple deleterious variants) modifying basic aspects of brain development. The lack of elevated autism symptoms in the majority of non-ASD children from single incidence families, particularly those without HLDAS, further supports the possibility that de novo events or rare recessive mutations of large effect may be driving autism in a significant proportion of single incidence families [11, 38].

The second novel finding was that ASD-affected children from multiple incidence families show the opposite pattern of their non-ASD sibling counterparts—*decreased* symptom burden. Thus, in multiple incidence families, non-ASD and ASD-affected children have closer levels of autism severity, whereas a wider spread of severity is seen in single incidence families. This pattern supports the presence of unique etiologic mixtures in single and multiple incidence families, although the exact nature of the differences in etiologic mixture cannot be discerned from symptom data. A recent study of common variants identified a stronger contribution of additive genetic variation in multiple incidence families [13], while studies of de novo CNVs have suggested enrichment in single incidence families [39, 40]. Subtle differences in the prevalence of common variants of small effect and de novo rare variants of large effect may be leading to small, but meaningful, differences in symptom aggregation between single and multiple incidence families. The present results highlight the importance of accounting for family incidence type in future etiologic investigations.

Third, HLDAS appears to be a major risk factor for elevated, but still sub-threshold, autism symptom levels [9]. Non-ASD children with HLDAS had substantially higher levels of autism symptoms (Δ18–21 SRS total raw score points) relative to non-ASD children without HLDAS, and elevations were present across both social and restricted/repetitive behavior domains. Given this pattern, non-ASD children with HLDAS, including those from single incidence families, may be at substantially greater risk of genetic transmission of autism. Prospective studies are needed to confirm this observation, examine rates of fecundity in these individuals, and estimate the risk of ASD in their offspring.

The final novel finding has increased autism symptom burden in next-born male siblings from multiple incidence female ASD-containing families and greater recurrence risk for all siblings in female ASD-containing families. This finding is congruent with Szatmari and colleagues [21] and the sex-specific threshold model. Under this model, females may require additional disease burden to cross threshold [23, 41, 42], with some females remaining sub-threshold [43] and possibly meeting DSM-5 criteria for social communication disorder or other developmental psychiatry disorders rather than autism spectrum disorder. It is important to note that previous studies lumping male and female non-ASD siblings from female ASD-containing families found a general increase in autism symptom burden. In the present study, the increase in symptom burden in siblings from female ASD-containing families was attenuated and non-significant when male and female non-ASD siblings were lumped. It is possible that previous significant findings using a lumped sibling group may have been driven by a larger increase in male non-ASD siblings. Alternatively, the present results may represent a false negative due to sampling variation in female non-ASD siblings. Regardless, the present findings raise the interesting possibility that all children from female ASD-containing families may be at higher recurrence risk, but residual symptom burden may be most apparent in non-ASD males from female ASD-containing families. If true, non-ASD females may be less likely than non-ASD males to exhibit the increase in risk behaviorally because of the presence of female-specific protective factors (e.g., social, hormonal, or genetic factors). Use of blinded, clinician-rated, quantitative instruments will be essential for clarifying the true pattern, ruling out the possibility of rater biases, and determining the exact nature of transmission of symptom elevations in pedigrees from female ASD-containing families.

### Limitations and future directions

The primary limitations of the present study were reliance on caregiver-reported symptoms and the use of data from an Internet-based registry. The IAN registry is carefully maintained with ease of reporting for parents. However, it is possible that some caregivers did not report data for an unaffected sibling. Many families in the registry contain multiple unaffected children and, if present, the most likely impact of under-reporting unaffected children is to reduce power to detect differences.

Caregiver-reported symptoms are susceptible to rater biases. For example, it is possible that the rater contrast between ASD-affected and non-ASD children in single incidence families is stronger and therefore leads caregivers to generate polarized ratings. However, this possibility seems unlikely to account for the observed pattern because: (1) the full range of symptom levels was observed in both single and multiple incidence families, (2) DSM-5 symptom increases in non-ASD children was stronger to symptom presentations seem more frequently in cognitively able individuals (B2: resistance to change and B3: restricted interests) rather than being a general increase across all symptoms and could not be accounted for by differential reliability, and (3) recurrence risks for categorical ASD diagnosis were consistent with symptom level findings. Rater contrast effects could also work in the opposite direction by reducing power and leading to underestimation of symptom level increases in non-ASD siblings from multiple incidence families. Thus, on balance, it seems unlikely that rater contrast effects generated the overall pattern of findings. Future studies that include clinician-rated symptoms, teacher-report data [11], or objective measurements of cognitive processes associated with the broad autism phenotype [7] will be helpful for further uncovering relationships between family structure or pedigree and phenotypic presentation.

Mapping of SRS and SCQ items to DSM-5 symptoms is a proxy for actual DSM-5 symptom ratings obtained as part of a comprehensive diagnostic evaluation. Future studies using DSM-5 symptom ratings will be essential to confirm the interesting pattern of symptom elevations in multiple incidence families. An additional limitation is that the IAN Internet registry is not representative of all families affected by ASD, due to over-inclusion of white non-Hispanic children and high socioeconomic status families, and does not include data from families unaffected by ASD. The presence of data from youth whose families are not affected by ASD might assist in interpreting the pattern of findings for non-ASD children from single and multiple incidence families. In spite of these limitations, the national scope and large size of the IAN registry—as well as the simultaneous collection of single and multiple incidence families—make it powerful for quantifying the effects of family structure on autism while covering the full range of autism symptom severity.

It is important to consider that the magnitude of family incidence and family sex effects in this study tended to be small in size. Effect sizes representing differences between single and multiple incidence families may be reduced due to stoppage—a decision to curtail future reproduction [44], without which a proportion of single incidence families would be re-classified as multiple incidence families. However, pedigree transmission studies identifying powerful genetic effects transmitted from unaffected mothers imply similar genetic mechanisms in at least a subset of single and multiple incidence families [45, 46]. Clearly, additional etiologic investigations are needed to sort out these mixtures, but the present results identifying relatively modest symptom differences suggests that etiologic mechanisms may differ in proportion but not in kind [39].

## Conclusions

With these limitations considered, the present findings replicated previous data identifying elevated autism symptoms in non-ASD children from multiple incidence families and extended these findings by showing the opposite pattern in ASD-affected children. Aggregation of autism symptoms was also influenced by the presence of the HLDAS phenotype in non-ASD youth and in families with an ASD-affected female, both of which increased burden. Non-ASD youth from multiple incidence families and youth with HLDAS appear to be at substantially elevated risk of autism transmission to their offspring. Finally, elevated symptom levels and recurrence risk in female ASD-containing families support the notion of sex-specific thresholds where females require greater genetic loading to meet ASD diagnostic criteria. Future large-scale studies that carefully ascertain family pedigrees and code for additional aspects of family structure (e.g., broad phenotype in biological parents) and include blinded clinician-rated and objective measures are needed to further clarify the genetic epidemiology of autism. These studies will be necessary for tailoring genetic counseling and for understanding background inherited liabilities that confer risk for clinical ASD in successive generations.

## Additional files

Additional file 1:
**Social Communication Questionnaire (SCQ) and Social Responsiveness Scale (SRS) item mapping to DSM-5 criteria.** This file provides the SCQ and SRS items which map to each of the DSM-5 criteria to create DSM symptom scores. Additional file 2:
**Main effect and interaction tests examining SRS total raw score by family incidence type and diagnostic status.** This file provides generalized estimating equation results for main effects and interactions examining autism symptom levels across single and multiple incidence families. Additional file 3:
**Frequency distributions of SRS total raw scores in non-ASD and ASD-affected children from single and multiple incidence families, separately for female and male children.** This file provides frequency distributions for SRS total raw scores by family incidence type and sex of the child. Additional file 4:
**Autism symptom levels (** ***M*** **+ 95 % CI) in non-ASD siblings (male and female siblings combined) from female ASD-containing and male-only families, separately for single and multiple incidence families.** This file provides SRS total raw scores (*M* +/−95 % CI) in all non-ASD children by family incidence type and family sex type. Additional file 5:
**Main effect and interaction tests examining SRS total raw score by family incidence type and family sex type in non-ASD siblings.** This file provides generalized estimating equation results for autism symptom levels across family incidence type and family sex type in non-ASD children. Additional file 6:
**Main effects examining SRS total raw score by family incidence type and family sex type in ASD-affected siblings.** This file provides generalized estimating equation results for autism symptom levels by family incidence type and family sex type in ASD-affected children. Additional file 7:
**Recurrence risk of ASD in next-born siblings by sex of the sibling and family sex type.** This file provides recurrence risk estimates for next-born siblings separately for male and female siblings and for male-only and female ASD-containing families.

## Abbreviations

ADHD: attention deficit hyperactivity disorder
ASD: autism spectrum disorder
DSM-5: Diagnostic and Statistical Manual of Mental Disorders—Fifth Edition
DSM-IV-TR: Diagnostic and Statistical Manual of Mental Disorders—Fourth Edition text revision
GEE: generalized estimating equations
HLDAS: history of language delay with autistic qualities of speech
IAN: Interactive Autism Network
IBM SPSS: International Business Machines Statistical Package for the Social Sciences
SCQ: Social Communication Questionnaire
SRS: Social Responsiveness Scale
