# Supplementary material for: Quantitative autism symptom patterns recapitulate differential mechanisms of genetic transmission in single and multiple incidence families
Source: Mol Autism. 2015 Oct 27;6:58. doi: 10.1186/s13229-015-0050-z (PMC4623917; doi:10.1186/s13229-015-0050-z)
Supplement: Additional file 1: — Social Communication Questionnaire (SCQ) and Social Responsiveness Scale (SRS) item mapping to DSM-5 criteria. This file provides the SCQ and SRS items which map to each of the DSM-5 criteria to create DSM symptom scores. [file 13229_2015_50_MOESM1_ESM.docx]

Additional File 1. Social Communication Questionnaire (SCQ) and Social Responsiveness Scale (SRS) item mapping to DSM-5 criteria.

**DSM-5**

|  | SCQ item # | SRS item # |
| --- | --- | --- |
| **Social Communication and Interaction** |  |  |
| 1: social-emotional reciprocity | 20, 28, 30, 31, 34, 37, 38, 40 | 13, 26, 38, 48 |
| 2: non-verbal communication | 9, 10, 22, 23, 24, 25, 26, 27, 33 | 45, 52, 55 |
| 3: relationships | 19, 36, 39 | 18, 37 |
| **Restricted, Repetitive Behavior** |  |  |
| 1: repetitive motor | 12, 15, 16 | 50 |
| 2: insistence on sameness | 8 | 24, 31, 61 |
| 3: restricted interests | 11, 13, 18 | 39 |
| 4: abnormal sensory | 14 | 20, 42, 63 |

Note: SCQ=Social Communication Questionnaire. SRS=Social Responsiveness Scale. This table depicts scale items that were mapped to DSM-5 criterion sets, preferentially drawing from the SCQ but eliminating items used to determine HLDAS, and incorporating SRS items as necessary to ensure adequate coverage of each criterion domain. Additional SRS items relate to the constructs encompassed by DSM-V but were not included to focus only on those with the clearest correspondence to criteria.
