# Supplementary material for: Quantitative autism symptom patterns recapitulate differential mechanisms of genetic transmission in single and multiple incidence families
Source: Mol Autism. 2015 Oct 27;6:58. doi: 10.1186/s13229-015-0050-z (PMC4623917; doi:10.1186/s13229-015-0050-z)
Supplement: Additional file 2: — Main effect and interaction tests examining SRS total raw score by family incidence type and diagnostic status. This file provides generalized estimating equation results for main effects and interactions examining autism symptom levels across single and multiple incidence families. [file 13229_2015_50_MOESM2_ESM.docx]

Additional File 2. Main effect and interaction tests examining SRS total raw score by family incidence type and diagnostic status.

|  | Wald X^2^ | DF | p |
| --- | --- | --- | --- |
| (Intercept) | 1641.6 | 1 | <.001 |
| Family incidence type | 0.3 | 1 | .574 |
| Diagnostic group (w/HLDAS)* | 2880.7 | 2 | <.001 |
| Age | 6.7 | 1 | .010 |
| Sex | 2.5 | 1 | .115 |
| Family incidence type by Diagnostic group | 17.3 | 2 | **<.001** |
| Family incidence type by sex | 1.3 | 1 | .246 |
| Diagnostic group by Sex | 0.5 | 2 | .770 |
| Family incidence type by Diagnostic group by Sex | <0.1 | 2 | .996 |

*Note: Diagnostic group includes separate codes for non-ASD with and without HLDAS. Bold designates significance of the key interaction term.
