# Supplementary material for: Quantitative autism symptom patterns recapitulate differential mechanisms of genetic transmission in single and multiple incidence families
Source: Mol Autism. 2015 Oct 27;6:58. doi: 10.1186/s13229-015-0050-z (PMC4623917; doi:10.1186/s13229-015-0050-z)

Additional File 3. Frequency distributions of SRS Total Raw scores in non-ASD and ASD-affected children from single and multiple incidence families, separately for female and male children.


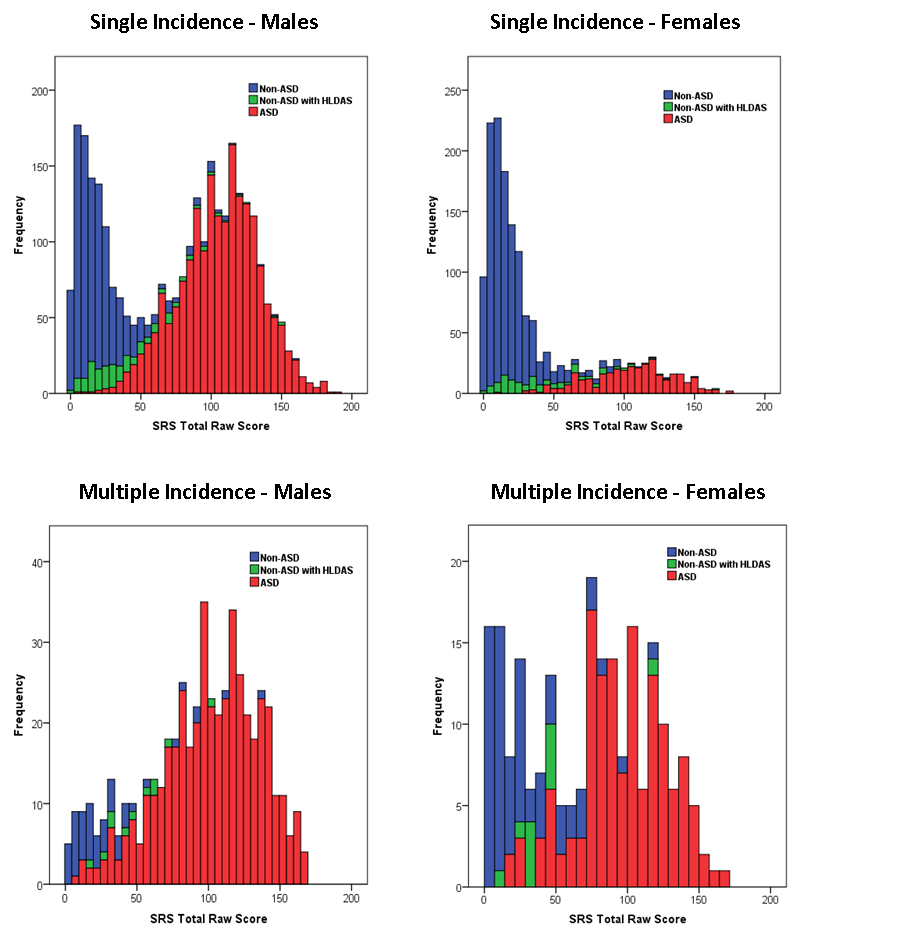

Supplement: Additional file 3: — Frequency distributions of SRS total raw scores in non-ASD and ASD-affected children from single and multiple incidence families, separately for female and male children. This file provides frequency distributions for SRS total raw scores by family incidence type and sex of the child. [file 13229_2015_50_MOESM3_ESM.docx]
