# Supplementary material for: Quantitative autism symptom patterns recapitulate differential mechanisms of genetic transmission in single and multiple incidence families
Source: Mol Autism. 2015 Oct 27;6:58. doi: 10.1186/s13229-015-0050-z (PMC4623917; doi:10.1186/s13229-015-0050-z)

Additional File 4. Autism symptom levels (M + 95% CI) in non-ASD siblings (male and female siblings combined) from female ASD-containing and male-only families, separately for single and multiple-incidence families.


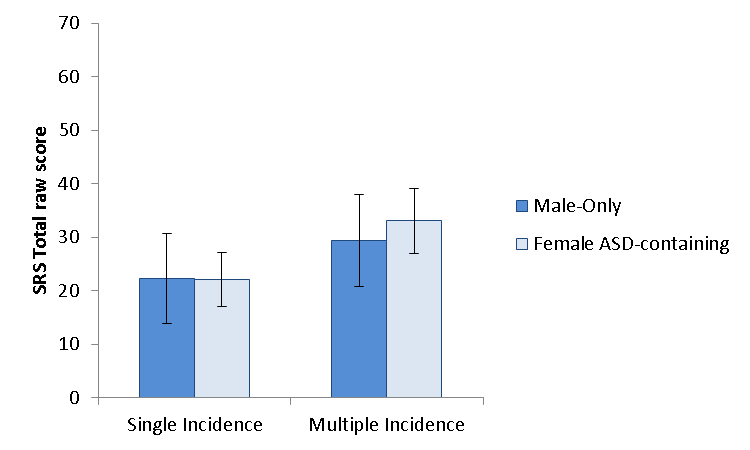

Supplement: Additional file 4: — Autism symptom levels ( M + 95 % CI) in non-ASD siblings (male and female siblings combined) from female ASD-containing and male-only families, separately for single and multiple incidence families. This file provides SRS total raw scores (M +/−95 % CI) in all non-ASD children by family incidence type and family sex type. [file 13229_2015_50_MOESM4_ESM.docx]
