# Supplementary material for: Quantitative autism symptom patterns recapitulate differential mechanisms of genetic transmission in single and multiple incidence families
Source: Mol Autism. 2015 Oct 27;6:58. doi: 10.1186/s13229-015-0050-z (PMC4623917; doi:10.1186/s13229-015-0050-z)
Supplement: Additional file 5: — Main effect and interaction tests examining SRS total raw score by family incidence type and family sex type in non-ASD siblings. This file provides generalized estimating equation results for autism symptom levels across family incidence type and family sex type in non-ASD children. [file 13229_2015_50_MOESM5_ESM.docx]

Additional File 5. Main effect and interaction tests examining SRS total raw score by family incidence type and family sex type in non-ASD siblings.

|  | Wald X^2^ | DF | p |
| --- | --- | --- | --- |
| (Intercept) | 289.8 | 1 | <0.001 |
| Family incidence type | 12.1 | 1 | <.001 |
| Family sex type | 0.4 | 1 | .511 |
| Age | 2.0 | 1 | .162 |
| Sex | 4.6 | 1 | .033 |
| Family incidence type by Sex | <0.1 | 1 | .972 |
| Family sex type by Sex | 8.6 | 1 | .003 |
| Family incidence type by family sex type | 0.6 | 1 | .439 |
| Family incidence type by Family sex type by Sex | 6.7 | 1 | **.009** |

Note. Bold designates significance of the key interaction term.
