# Supplementary material for: Quantitative autism symptom patterns recapitulate differential mechanisms of genetic transmission in single and multiple incidence families
Source: Mol Autism. 2015 Oct 27;6:58. doi: 10.1186/s13229-015-0050-z (PMC4623917; doi:10.1186/s13229-015-0050-z)
Supplement: Additional file 6: — Main effects examining SRS total raw score by family incidence type and family sex type in ASD-affected siblings. This file provides generalized estimating equation results for autism symptom levels by family incidence type and family sex type in ASD-affected children. [file 13229_2015_50_MOESM6_ESM.docx]

Additional File 6. Main effects examining SRS total raw score by family incidence type and family sex type in ASD-affected siblings.

|  | Wald X^2^ | DF | p |
| --- | --- | --- | --- |
| (Intercept) | 3700.3 | 1 | <0.001 |
| Family incidence type | 12.1 | 1 | <.001 |
| Family sex type | 5.5 | 1 | **.020** |
| Age | 8.6 | 1 | .003 |
| Sex | 9.9 | 1 | .002 |

Note. Bold designates significance of the key main effect.
