# Supplementary material for: Quantitative autism symptom patterns recapitulate differential mechanisms of genetic transmission in single and multiple incidence families
Source: Mol Autism. 2015 Oct 27;6:58. doi: 10.1186/s13229-015-0050-z (PMC4623917; doi:10.1186/s13229-015-0050-z)
Supplement: Additional file 7: — Recurrence risk of ASD in next-born siblings by sex of the sibling and family sex type. This file provides recurrence risk estimates for next-born siblings separately for male and female siblings and for male-only and female ASD-containing families. [file 13229_2015_50_MOESM7_ESM.docx]

Additional File 7. Recurrence risk of ASD in next-born siblings by sex of the sibling and family sex type.

|  | ASD  N | Non-ASD  N | Next born siblings  N | % | 95% CI | RR | P |
| --- | --- | --- | --- | --- | --- | --- | --- |
| Next Born Males | 429 | 1518 | 1947 | 22.0% | 20.3%-23.9% | 2.56 (2.15-3.07) | <.001 |
| Next Born Females | 149 | 1584 | 1733 | 8.6% | 7.4%-10.0% |  |  |
| Male-Only Families | 351 | 2610 | 2961 | 11.9% | 10.7%-13.1% | 2.66 (2.29-3.09) | <.001 |
| Female-Containing Families | 227 | 492 | 719 | 31.6% | 28.3%-35.1% |  |  |

Note. 95% CI denotes the 95% confidence interval around each recurrence %. RR=Relative Risk.
